# Fayette County Medical Society

**Published:** 1857-07

**Authors:** A. D. Stearns, F. B. Haller

**Affiliations:** President; Secretary


### *Fayette County Medical Society.*

Pursuant to a call of the physicians of Fayette county, a meeting was held at the office of Dr. Wilkins on the evening of April 1st, for the purpose of organizing a County Medical Society.

Dr. Stearns was called to the chair, and Dr. Haller appointed Secretary.

A Constitution and By-Laws, for the government of the Society, were presented, and adopted.

The following gentlemen were then elected officers for the ensuing year:

Dr. A. D. Stearns, *President.*

Dr. Thomas Wilkins, *Vice-President.*

Dr. F. B. Haller, *Secretary.*

Dr. E. W. Boothe, *Treasurer.*

A list of charges for professional services was presented, and unanimously adopted.

On motion, it was

*Resolved*, That the Fee-Bill be printed, with the names of the members of the Society appended thereunto, and that every member be required to post a bill conspicuously in his office.

The Society then adjourned to meet in monthly session on the first Wednesday in August, at 8 o'clock P. M.

A. D. STEARNS, M. D., President.

F. B. HALLER, M. D., Secretary.

